# Supplementary material for: Unwinding of a DNA replication fork by a hexameric viral helicase
Source: Nat Commun. 2021 Sep 20;12:5535. doi: 10.1038/s41467-021-25843-6 (PMC8452682; doi:10.1038/s41467-021-25843-6)
Supplement: Supplementary file 1 — Supplementary Information [file 41467_2021_25843_MOESM1_ESM.pdf]

Unwinding of a DNA replication fork by a hexameric viral helicase

Abid Javed[1#], Balazs Major[2#], Jonathan A. Stead[2#], Cyril M. Sanders[2*], Elena V. Orlova[1*]

[1]Department of Biological Sciences, Birkbeck College, Institute of Structural and Molecular Biology, Malet Street, London WC1E 7HX, United Kingdom
[2]Academic Unit of Molecular Oncology, Department of Oncology and Metabolism, University of Sheffield, Medical School, Beech Hill Rd., Sheffield, S10 2RX, UK

[#] These authors contributed equally

* Corresponding authors: Elena V. Orlova
E-mail: e.orlova@mail.cryst. bbk.ac.uk,

Cyril M. Sanders
E-mail: c.m.sanders@sheffield.ac.uk

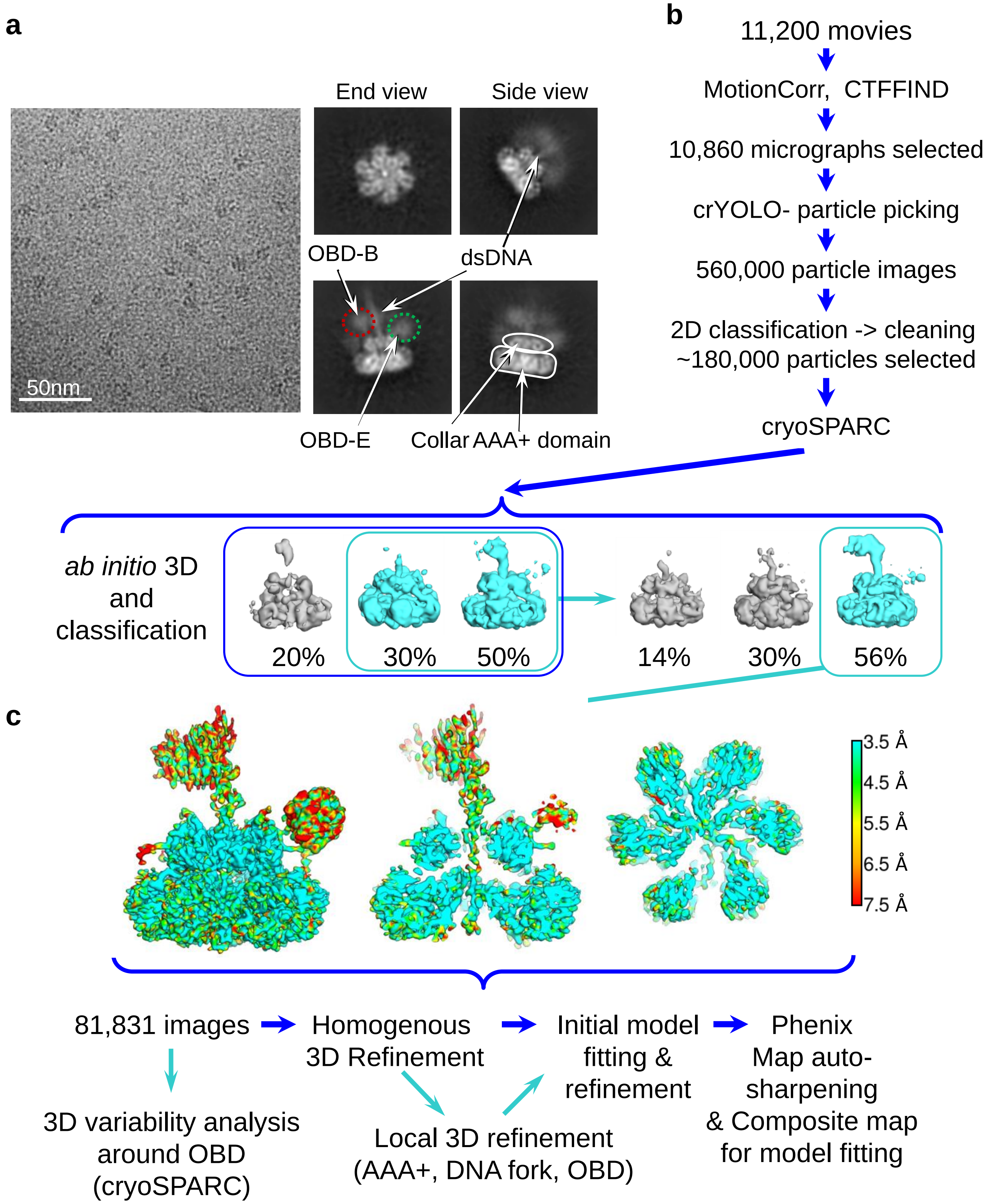
a
End view
Side view
50nm
OBD-B
dsDNA
OBD-E
Collar AAA+ domain
b
11,200 movies
MotionCorr, CTFFIND
10,860 micrographs selected
crYOLO- particle picking
560,000 particle images
2D classification -> cleaning
~180,000 particles selected
cryoSPARC
ab initio 3D and classification
20%
30%
50%
14%
30%
56%
c
3.5 Å
4.5 Å
5.5 Å
6.5 Å
7.5 Å
81,831 images
Homogenous 3D Refinement
Initial model fitting & refinement
Phenix Map auto-sharpening & Composite map for model fitting
3D variability analysis around OBD (cryoSPARC)
Local 3D refinement (AAA+, DNA fork, OBD)

Supplementary Fig. 1 Overall diagram of the image processing protocol. a. A representative cryo-EM micrograph of the E1RF complex. **b**. The workflow of the cryo-EM image analysis, blue arrows link the steps from data collection to 3D reconstructions. The grey and cyan outlined boxes show 3D classes, the corresponding percentage of particle images comprising each class are shown below the classes. The refined map of the E1RF complex was calculated using ~80,000 particle images. **c**. The EM map of the E1RF complex coloured according to local resolution. The map is unsharpened and masked including OBD B and OBD E. Regions at higher resolution are in cyan, regions at lower in resolution are in orange: The left panel shows the outer surface of the map and the middle panel demonstrate the local resolution within a central slice. The right panel represent the local resolution within a section through the central part of the AAA+ domain. The bar on the right indicates the correspondence of colours in maps to the resolution in Angstroms. The final steps for 3D reconstruction and sharpening of the map used for atomic modelling are indicated at the bottom of the scheme.

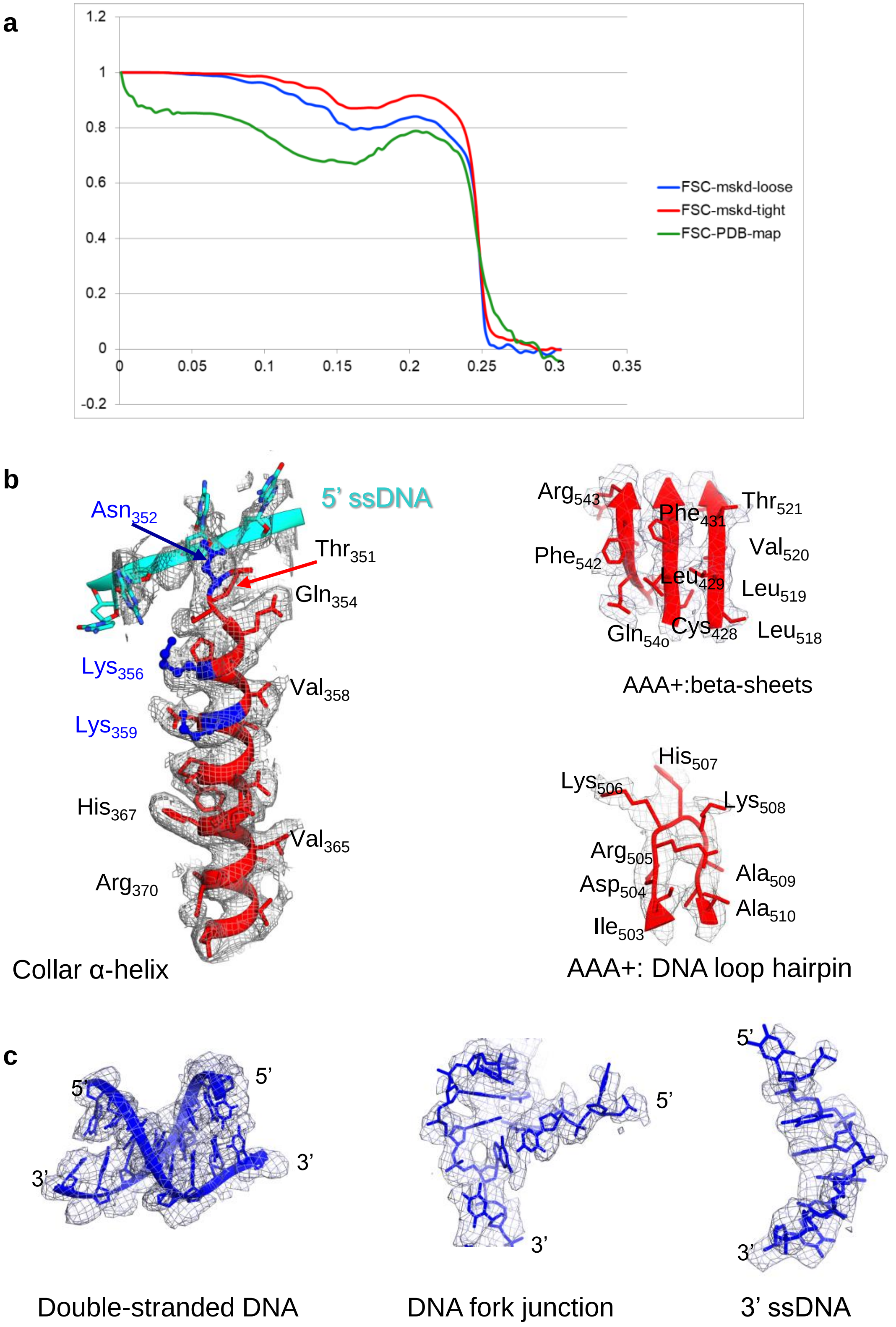
a
1.2
1
0.8
0.6
0.4
0.2
0
-0.2
0
0.05
0.1
0.15
0.2
0.25
0.3
0.35
FSC-mskd-loose
FSC-mskd-tight
FSC-PDB-map
b
Asn352
5' ssDNA
Thr351
Gln354
Lys356
Val358
Lys359
His367
Val365
Arg370
Collar α-helix
Arg543
Phe431
Thr521
Phe542
Val520
Leu429
Leu519
Gln540
Cys428
Leu518
AAA+:beta-sheets
His507
Lys506
Lys508
Arg505
Asp504
Ala509
Ala510
Ile503
AAA+: DNA loop hairpin
c
5'
5'
3'
3'
5'
3'
5'
3'
Double-stranded DNA
DNA fork junction
3' ssDNA

Supplementary Fig. 2 Assessment of map resolution. a. Fourier shell correlation (FSC) curves for the E1RF map. The curve in blue is for the map using a loose mask, the red curve represents the FSC of the map within a tight mask. The curve in green is between the cryo-EM map within a loose mask, and the generated, fitted atomic model. **b.** Fitting of the atomic models into the EM densities. The panel shows fitted secondary structural elements (in red) of the E1 helicase into the EM density (grey mesh). A fitted α-helix of the collar domain (left panel) with a portion of 5´ ssDNA (in cyan), region of β-strands (middle) and a DNA binding β-hairpin (right) with relevant amino acids labelled. **c.** Fitting of DNA (in blue) into the E1RF EM densities; double stranded DNA (left), the DNA fork junction and 5´ ssDNA (middle) and the 3´ ssDNA (right panel).

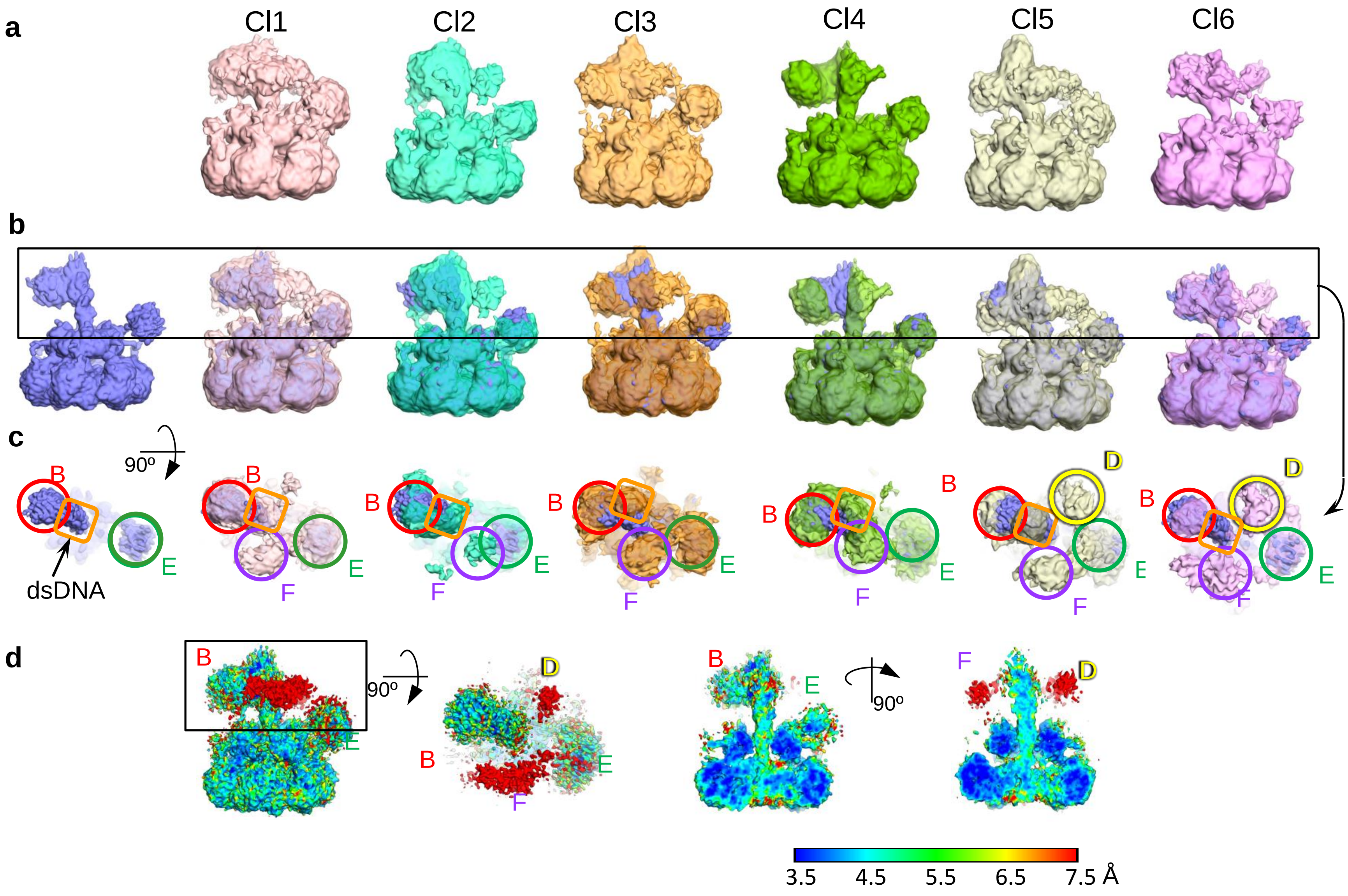

a
Cl1
Cl2
Cl3
Cl4
Cl5
Cl6
b
c
90º
B
E
F
D
dsDNA
d
3.5
4.5
5.5
6.5
7.5 Å

Supplementary Fig. 3 Variations in the positions of the OBD domains in the E1RF structure. a. Side views of 3D maps: Class 1 in light pink, Class 2 in cyan and Class 3 in light orange, Class 4 in green, Class 5 in beige, and Class 6 in magenta. **b**. The very left panel shows the overall refined 3D E1RF map at a resolution of 3.9 Å (in blue). The following panels show superposition of the classes (in **a**) with the refined map. The black box outlines the OBD regions shown from the top in **c**. **c**. The positions adopted by the OBDs in classes 1-6 are demonstrated. The OBDs corresponding to subunits B, D, E, and F are shown within red, yellow, green, and purple circles respectively. The dsDNA region is shown in an orange box. **d.** Local resolution map of the E1RF complex before 3D classification. The left panel demonstrates the local resolution of the unmasked map including all OBD domains. The second panel from the left shows the local resolution map from the top. The OBDs B and E are well defined, while the locations of F and E are indistinct. The two right panels show central perpendicular sections of the map (along the central axis of the E1HD) indicating the lower resolution of the F and D OBD domains. The bar at the bottom indicates the correspondence of the colour in maps to the resolution in Angstroms.

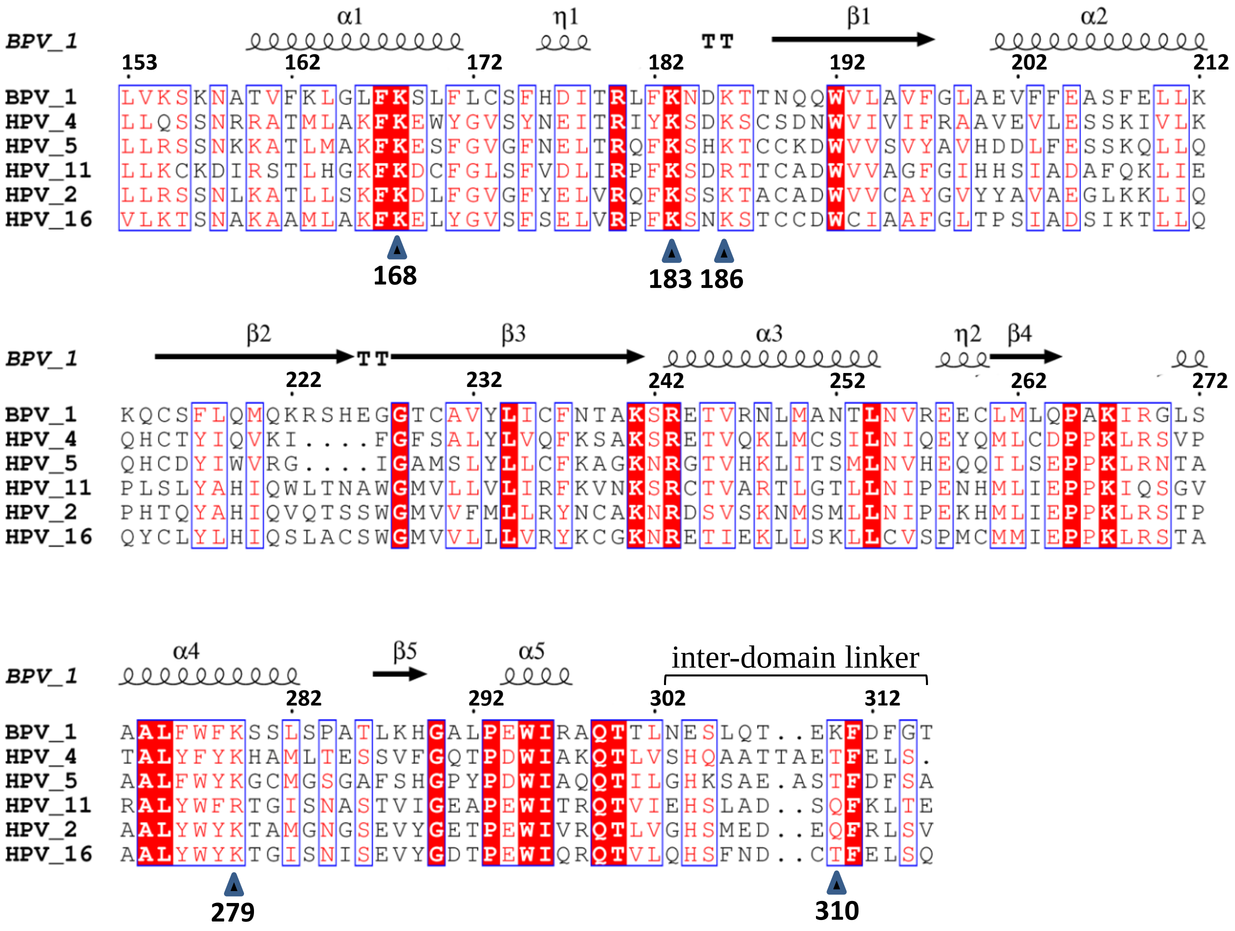

Supplementary Fig. 4 E1 sequence alignment. Alignment of the E1 OBD and interdomain linker region sequences from representative of the major papillomavirus genera: Alpha-papillomavirus, HPV 2 [ABO14922.1], HPV 11 [AAA46929.1] and HPV 16 [NP_041327.2]; beta-papillomavirus, HPV 5 [AFL02855.1]; delta papillomavirus, BPV 1 [NP_056739.1] and gamma-papillomavirus, HPV 4 [NP_040891.1]. The numbering of residues is for BPV1 E1 and the residues mutated in this study are indicated. The alignment and secondary structure for the BPV E1 OBD (top) were generated with ESPript 3.0 (Robert, X. and Gouet, P. (2014) Deciphering key features in protein structures with the new ENDscript server. *Nucleic Acids Res.* 42(W1), W320-W324. doi: 10.1093/nar/gku316).

a

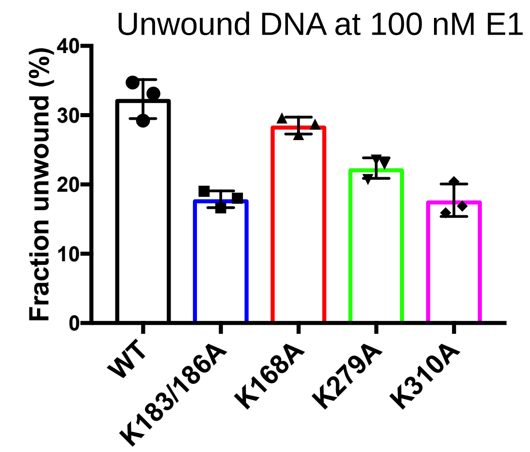

b

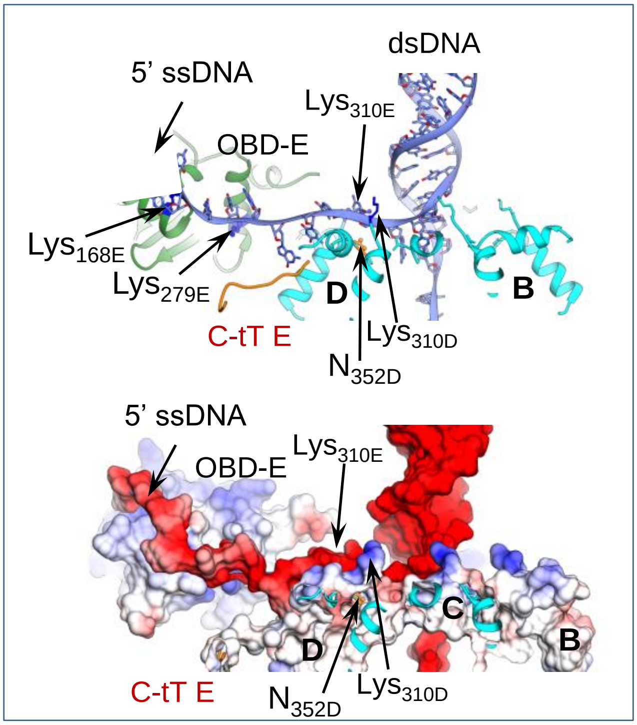

c

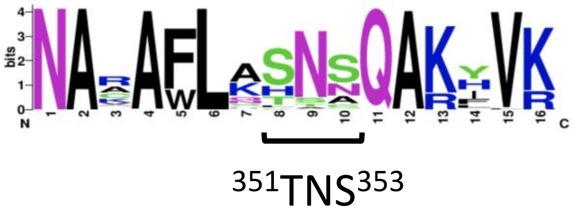

d

	k_{cat}(s^{-1})	k^{rel}
**Wild-type**	0.383±0.014	1
**N352K**	0.357±0.024	0.93
**N352Y**	0.592±0.01	1.54
**N352D**	0.463±0.015	1.2
**N352F**	0.362±0.014	0.95
**N352G**	0.324±0.016	0.84

e

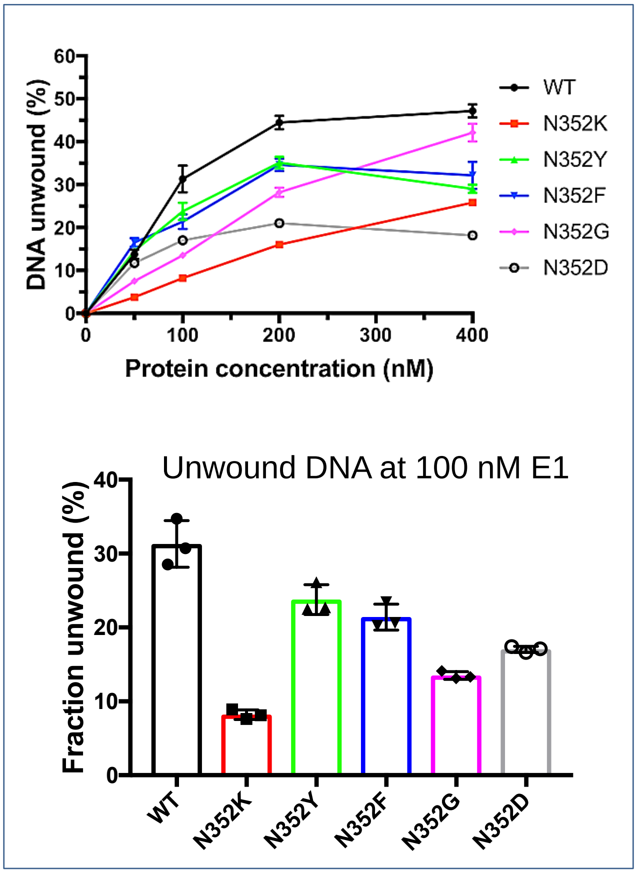

f

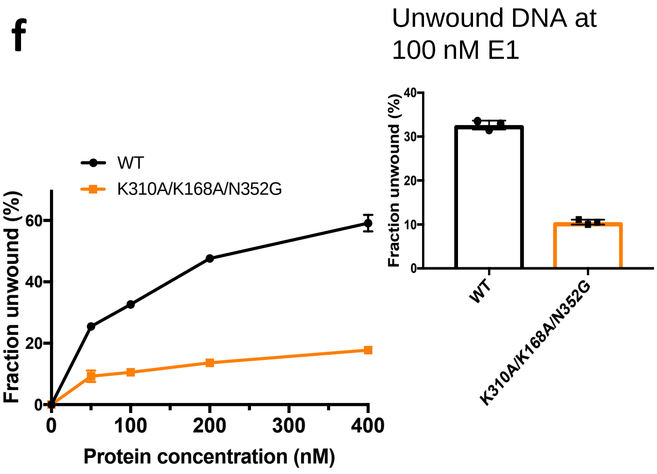

Supplementary Fig. 5 The path of the 5´ ssDNA. **a**. For comparison, unwinding activity at 100 nM (n = 3 independent experiments) for the mutants (wild type (WT) is shown in black; K183A/K186A in blue; K168A in red; K279Ain green; K310A in magenta; mean values are shown with error bars ± SD) in the interdomain linker and OBD domain shown in Fig. 4 (main manuscript). **b**. Representation of the electrostatic charges of the E1RF collar domains and 5´ ssDNA. The upper panel shows a cross-section of the atomic model of E1RF at the level of the 5´ ssDNA on the top of the collar domain. The bottom panel shows the electrostatic potential of the ssDNA and the upper part of the collar. Positively charged regions of the molecular surface of collar domain are shown in blue and negatively charged regions in red. The negative charged ssDNA is located in a grove between collar subunits, the TNS loop of subunit D and Lys310 of subunit E are in a close proximity to ssDNA, as shown in the bottom panel. The OBD-E has regions of positive charge on the surface for interaction with the 5´ ssDNA. The electrostatics indicate that while the ssDNA assumes a path that is predominantly neutral in character, specific positive points (Lys310 in the inter-domain linker, the surface of OBD E and the polar residues of the TNS loop) act to fix and guide the 5´ ssDNA path. The C-terminal tails (C-tT) are positioned ~8 Å below the ssDNA. The segment shown is for residues 584-594, of which 9 of the 11 residues are either glutamate or aspartate. The proximity of this segment to the ssDNA indicates repulsion between these electronegative elements, thus ensuring unimpeded passage of the ssDNA away from the helicase domain subassembly. **c**. Representative sequence logo showing the amino acid conservation in the collar domain area of the E1 helicase domain, residues 344-359. **d**. ATP*ase* activity (k_{cat}) of helicase domain substitutions. k_{cat} of Asn352 substitutions relative to wild type E1(k^{rel}) determined in the absence of DNA. **e**. Helicase activity of wild type E1 and Asn352 substitutions. The N352K substitution demonstrates the most significant defect in unwinding (~25% of wild-type) while the DNA-independent ATP*ase* activity is similar to wild-type (k^{rel} 0.93). The graph shows the data for three independent helicase experiments (50, 100, 200 and 400 nM E1; 0.1 nM fork substrate); mean values are shown with error bars ± SD. Unwinding activity at 100 nM E1 is shown in the bar chart below (n = 3 experimentally independent data points; mean values are shown with error bars ± SD. Graph lines and the bars in the chart are indicated by colours: wild type (WT)in black; N352K in red; N352Y in green; N352F in blue; N352G in magenta and N352D in grey. **f.** Unwinding activity of the triple mutant K310A/K168A/N352G used to probe interactions with the 5′ passive ssDNA strand. In the graph and the bar charts the wild type is indicated in black and the triple mutant K310A/K168A/N352G in orange. Results are shown as mean values with error bars ± SD. Note, errors in some measurements (central part of the graph) are very small and are within the size of symbols used in the graph.

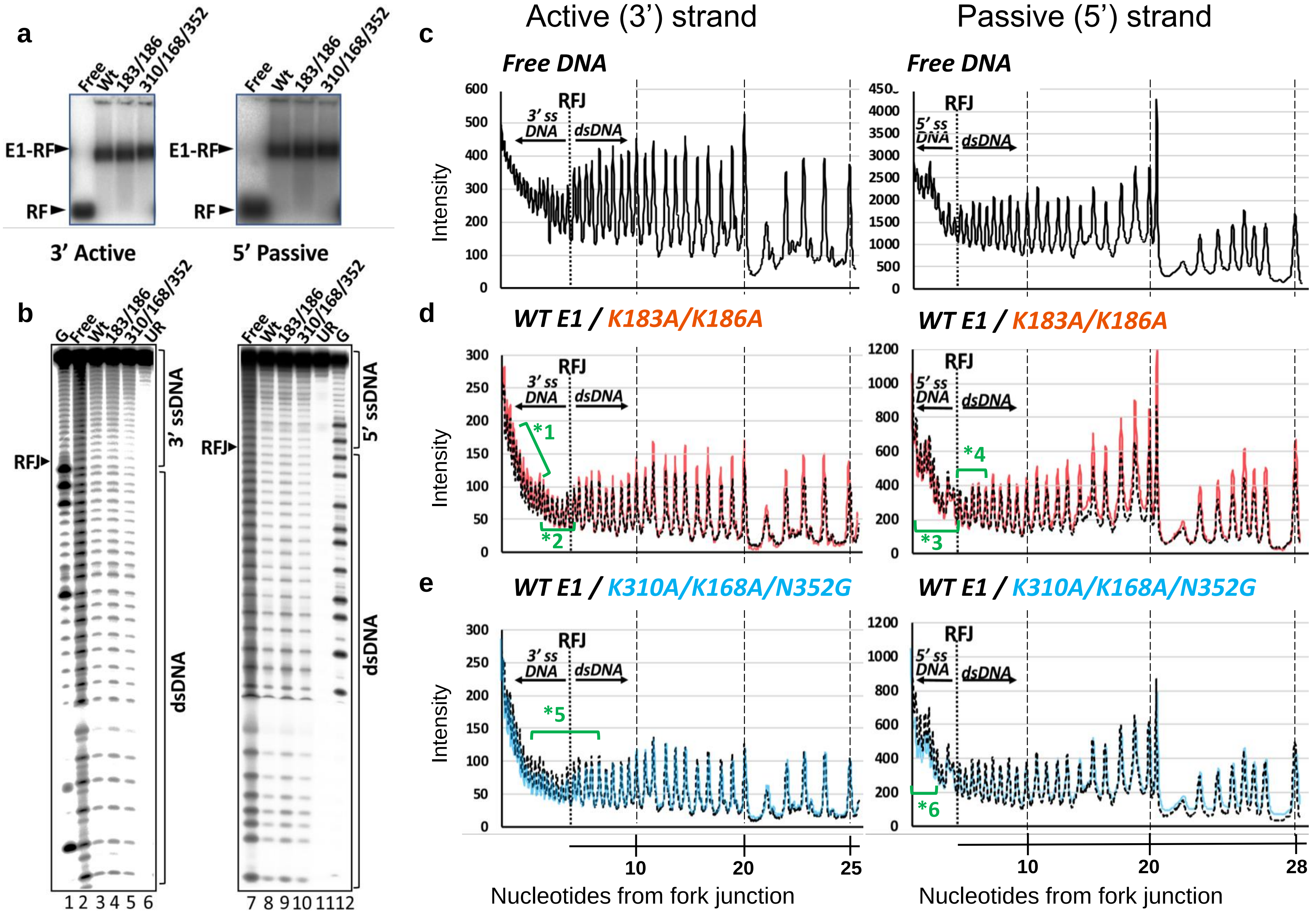

a
Free
Wt
183/186
310/168/352
E1-RF
RF
3' Active
5' Passive
b
G
UR
RFJ
3' ssDNA
5' ssDNA
dsDNA
1 2 3 4 5 6
7 8 9 10 11 12
c
Active (3') strand
Passive (5') strand
Free DNA
Intensity
RFJ
3' ss DNA
5' ss DNA
dsDNA
d
WT E1 / K183A/K186A
*1
*2
*3
*4
e
WT E1 / K310A/K168A/N352G
*5
*6
Nucleotides from fork junction
10
20
25
28

Supplementary Fig. 6 E1RF hydroxyl radical (OH•) DNA footprinting. E1-RF complexes were assembled with E1 K183A/K186A to probe interaction of OBD B with the dsDNA and E1 K310A/K168A/N352G to probe interactions with the 5´ ssDNA of RF. Complete DNA binding was confirmed by gel-shift analysis (**a**, RF - replication fork DNA, E1-RF is the wild-type (Wt)/variant E1 helicase-RF complex). **b**. OH• cleavage products analysed on sequencing gels and exposed to phosphor imaging plates (G - guanine ladder, Free - unbound DNA and UR- unreacted substrate). **c.** Phosphor imaging densitometry traces of the OH• cleavage ladders for free DNA (lanes 2 and 7 in **b**). **d**. The traces for wild-type E1RF (dashed line, black) overlaid with traces for the variant K183A/K186A (in orange). For the active strand, lane 3 is compared to 4 in **b**, and for the passive strand lane 8 is compared to 9. *1 indicates the region of 3´ active strand ssDNA with increases in peak heights of up to ~12% for K183A/K186A. Note, these changes are reported for the ~9 ss DNA nucleotides closest to the unwinding point. The longer DNA molecules beyond this segment are less well resolved in the gel and density traces are more difficult to align. Furthermore, these ssDNA residues are beyond the segment of 3´ active strand ssDNA interacting with the β-hairpins in the AAA+ motor domain and are likely to have exited the motor domain. *2 indicates the region close to the RFJ with near identical susceptibility to OH• cleavage, between the Wt and variant complex. *3 highlights the 5´ ssDNA of RF where protection is near-equivalent between Wt and variant E1-RF. *4 highlights the dsDNA region in the passive strand with near equivalent protection between Wt and variant RF complexes. **e**. Traces for K310A/K168A/N352G (in blue) compared to wild-type (dashed line, black), lane 3 compared to 5 and lane 8 compared to 10, active and passive strand respectively in **b.** *5, the region around the RFJ showing increased protection in E1 K310A/K168A/N352G-RF compared to wild-type E1RF. *6, region of enhanced protection in the 5´ ssDNA of E1 K310A/K168A/N352G-RF. The entire experimental series was repeated three times and showed that the same general features described above are preserved.

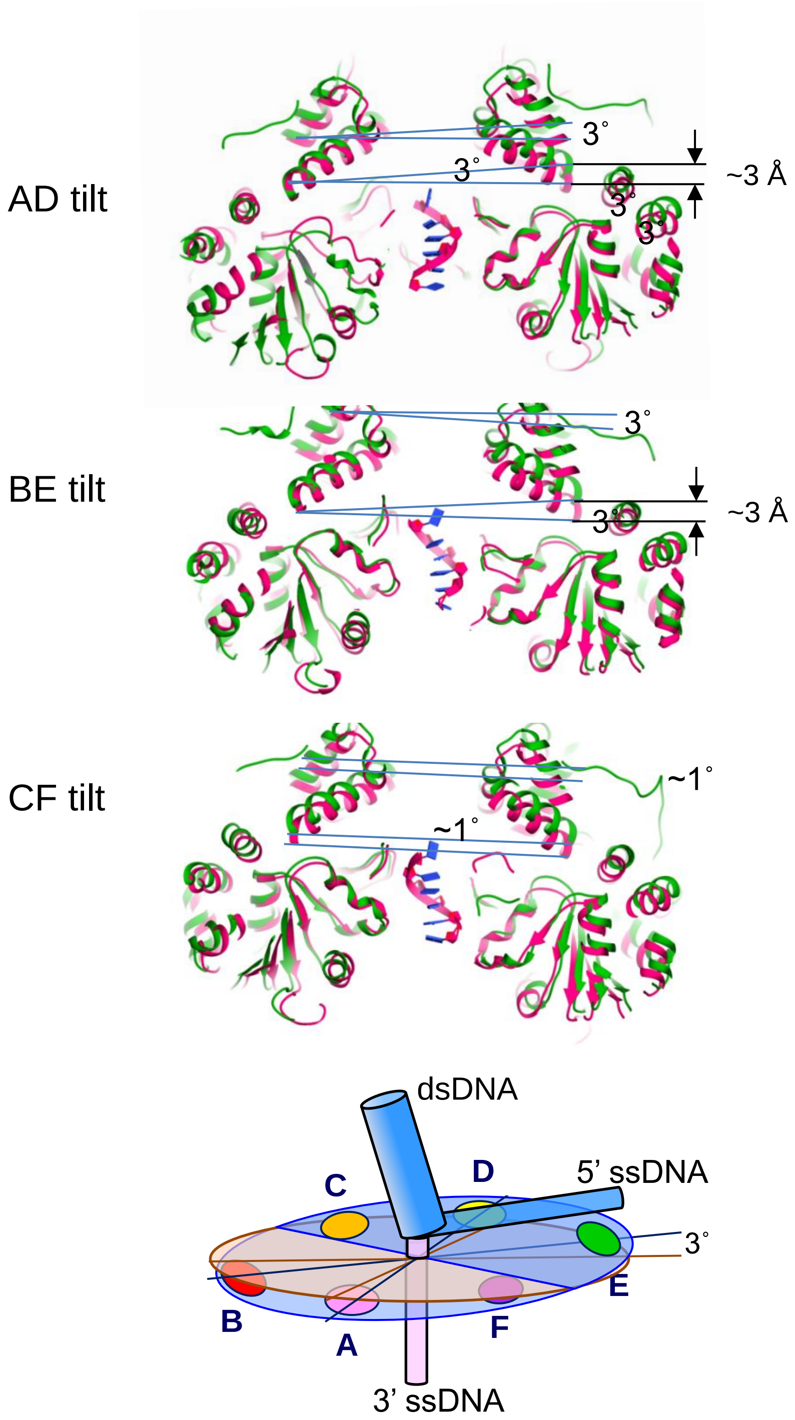

Supplementary Fig. 7 Deviations in positions of the collar domain between E1RF and the X-ray structure of E1HD with ssDNA and ADP bound. Tilt angles between corresponding pairs of subunits are shown. Pairs AD and BE have similar tilts, while the CG pair is hardly tilted, indicating that the axis of the tilt goes in the direction perpendicular to the direction of the 5' ssDNA across the top of the collar ring. The tilt of the collar is shown schematically in the bottom panel.

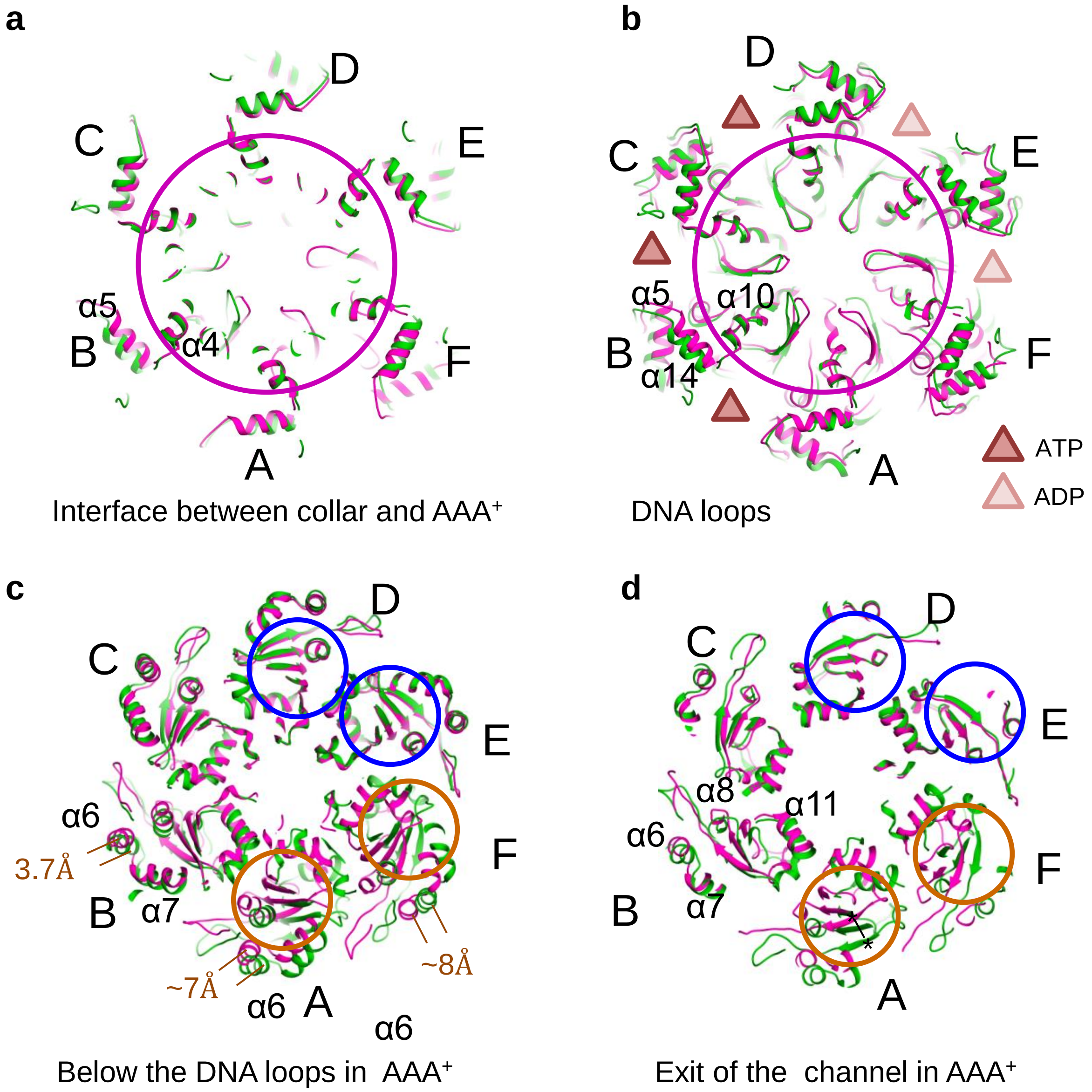
a
D
C
E
α5
α4
B
F
A
Interface between collar and AAA+
b
D
C
E
α5
α10
B
α14
F
A
ATP
ADP
DNA loops
c
D
C
E
α6
3.7Å
F
B
α7
~7Å
~8Å
α6
A
α6
Below the DNA loops in AAA+
d
D
C
E
α8
α11
α6
F
B
α7
A
Exit of the channel in AAA+

Supplementary Fig. 8 Conformational deviations between E1RF and the X-ray structure of E1HD with ssDNA and ADP bound **a**. A cross-section of the E1RF complex, showing the interface area between the collar domain and the AAA+ domain. The X-ray model (PDB 2GXA in magenta) and cryo-EM atomic model (in green) are fitted into cryo-EM map. The magenta circle indicates a central area with good superposition between the structures. **b**. The region of the DNA binding b-hairpins in the aligned atomic models of the X-ray (in magenta) and cryo-EM (in green) structures. The nucleotide states at the subunit interfaces, as reported in the X-ray structure, are indicated by brown (ATP) or pale brown (ADP) triangles. The central region of the DNA binding β-hairpins that interact with the 3´ ssDNA are shown in the magenta circle. There is excellent alignment between hairpins C, D, E and F, a minor discrepancy between the Bs and a significant shift of the A hairpins (see Figure 6, main manuscript) from each structure. **c**. The AAA+ domains in the aligned models in the area below the DNA loops. Subunits D and E demonstrate good consistency between the two structures (circled in blue). Displacements between secondary structure elements in the AAA+ domain of the X-ray and the EM structures of subunits A and F are indicated in the brown circles. Distances for the displacements of helix α6 (~7 Å), at the periphery of the complex, are indicated. **d**. A slice of the AAA+ domains at the bottom of the helicase DNA channel where ssDNA exits. Large displacements in the AAA+ secondary structural elements are seen between the X-ray structure and the EM structure for subunits A and F (brown circles). Intermediate displacements are seen in subunits B and C, while D and E show good alignment.

Supplementary Table 1. Cryo-EM analysis of E1RF: data collection, refinement and validation statistics.

**E1-FL-Fork**	
EMDB	11852
PDB	7APD
Data Collection and processing	
Microscope	Titan Krios (300kV)
Pixel size (Å/pix.)	1.085
Camera	K3
Electron Dose (e-/A2)	45
Defocus range (µm)	(-1.2 to -3.0)
Symmetry imposed	C1
Software	cryoSPARC v2.1, RELION 3.0
Initial particle images (no.)	560,000
Final particle images (no.)	81,831
Map Resolution (Å, 0.143 criterion)	3.85
Map Resolution Range (Å)	3.5 – 8.0
Sharpening B factor ($Å^2$)	-102.1
Model Refinement & Validation	
Initial models used (PDB code)	2GXA, 1KSY
Model composition	
Non-Hydrogen atoms	17307
Protein and DNA residues	2020; 76
Ligands	0
R.m.s. deviations	
Bond lengths (Å)	0.006
Bond angles (°)	1.08
Validation	
Molprobity score	2.3
All-atom Clashscore	19.2
Poor rotamers (%)	0
Ramachandran plot	
Outliers	0.15
Favoured %	91.5
Allowed %	8.4
