# Supplementary material for: Unwinding of a DNA replication fork by a hexameric viral helicase
Source: Nat Commun. 2021 Sep 20;12:5535. doi: 10.1038/s41467-021-25843-6 (PMC8452682; doi:10.1038/s41467-021-25843-6)
Supplement: Supplementary file 3 — Description of Additional Supplementary Files [file 41467_2021_25843_MOESM3_ESM.pdf]

Description of Additional Supplementary Files

File Name: Supplementary Movie 1

Description: **Structural transition between subunits of the E1 helicase.**

Structural transition between subunits (A to B, B to C and so forth) are modelled to show the conformational changes within the individual subunits that accompany the conformational wave around the helicase ring. Atomic models of the E1 helicase domain are fitted in the E1RF EM map and coloured according to the convention used throughout the manuscript. The movie highlights the interactions of the DNA binding β-hairpins linked to 3′ ssDNA translocation and the motion of the α-5 'hinge' that coordinates movements between the collar and AAA+ domains

File Name: Supplementary Movie 2

Description: **Reconstruction of the E1 helicase**.

The E1RF cryo-EM map is coloured with the E1 subunit A of the hexamer in magenta, B in red, C in orange, D in yellow, E in green and F in purple. The bound DNA replication fork is shown in cyan, along with OBD-B (in red) and OBD-E (green) with fixed positions in the structure. The complex is viewed from the side, top (with the incoming dsDNA) and bottom. The atomic models of the E1 helicase domain, OBD and the DNA fork are shown fitted into the cryo-EM map, represented as a semi-transparent surface. Finally, a central cross-section shows the organisation of the replication fork and protein-DNA interactions. OBD-B is located in the close proximity to the dsDNA, the passive 5′ ssDNA passes over the top of the collar domain and interacts with OBD-E, while the 3′ active ssDNA goes through the central channel of the hexamer. Interactions with the DNA binding b-hairpins are observed in the AAA motor domain.
