# Supplementary material for: Unwinding of a DNA replication fork by a hexameric viral helicase
Source: Nat Commun. 2021 Sep 20;12:5535. doi: 10.1038/s41467-021-25843-6 (PMC8452682; doi:10.1038/s41467-021-25843-6)
Supplement: Supplementary file 6 — Reporting Summary [file 41467_2021_25843_MOESM6_ESM.pdf]

nature research

Corresponding author(s): E.V. Orlova

Last updated by author(s): Aug 3, 2021

Reporting Summary

Nature Research wishes to improve the reproducibility of the work that we publish. This form provides structure for consistency and transparency in reporting. For further information on Nature Research policies, see our Editorial Policies and the Editorial Policy Checklist.

Statistics

For all statistical analyses, confirm that the following items are present in the figure legend, table legend, main text, or Methods section.

n/a	Confirmed	
☐	☒	The exact sample size (*n*) for each experimental group/condition, given as a discrete number and unit of measurement
☐	☒	A statement on whether measurements were taken from distinct samples or whether the same sample was measured repeatedly
☒	☐	The statistical test(s) used AND whether they are one- or two-sided<br>*Only common tests should be described solely by name; describe more complex techniques in the Methods section.*
☒	☐	A description of all covariates tested
☒	☐	A description of any assumptions or corrections, such as tests of normality and adjustment for multiple comparisons
☐	☒	A full description of the statistical parameters including central tendency (e.g. means) or other basic estimates (e.g. regression coefficient) AND variation (e.g. standard deviation) or associated estimates of uncertainty (e.g. confidence intervals)
☒	☐	For null hypothesis testing, the test statistic (e.g. *F*, *t*, *r*) with confidence intervals, effect sizes, degrees of freedom and *P* value noted<br>*Give P values as exact values whenever suitable.*
☒	☐	For Bayesian analysis, information on the choice of priors and Markov chain Monte Carlo settings
☒	☐	For hierarchical and complex designs, identification of the appropriate level for tests and full reporting of outcomes
☒	☐	Estimates of effect sizes (e.g. Cohen's *d*, Pearson's *r*), indicating how they were calculated

Our web collection on statistics for biologists contains articles on many of the points above.

Software and code

Policy information about availability of computer code

Data collection	EPU (Thermo Fischer Scientific)
Data analysis	Relion v3.0, MotionCor 2 v1.4.2, Cttfind 4.1, cryoSPARC v2.1, COOT v8.0, UCSF Chimera v1.14, Phenix v1.18

For manuscripts utilizing custom algorithms or software that are central to the research but not yet described in published literature, software must be made available to editors and reviewers. We strongly encourage code deposition in a community repository (e.g. GitHub). See the Nature Research guidelines for submitting code & software for further information.

Data

Policy information about availability of data

All manuscripts must include a data availability statement. This statement should provide the following information, where applicable:

- Accession codes, unique identifiers, or web links for publicly available datasets
- A list of figures that have associated raw data
- A description of any restrictions on data availability

The data required for assessment and validation of conclusions in the paper are described within the main text or supplementary section of the paper. CryoEM map for E1-RFJ has been deposited and is available at the Electron Microscopy Databank (www.ebi.ac.uk/pdbe/emdb) and can be accessed using accession code EMD-11852. The atomic models used for the fitting and analysis of the cryoEM map has been deposited to the Protein Data Bank (www.rcsb.org) and can be accessed using accession code PDB:7APD.

Field-specific reporting

Please select the one below that is the best fit for your research. If you are not sure, read the appropriate sections before making your selection.

☒ Life sciences ☐ Behavioural & social sciences ☐ Ecological, evolutionary & environmental sciences

For a reference copy of the document with all sections, see nature.com/documents/nr-reporting-summary-flat.pdf

Life sciences study design

All studies must disclose on these points even when the disclosure is negative.

Sample size	For E1-RFJ complex analysis, cryoEM dataset consisting of several thousand micrographs were collected. The number of micrographs (determining the sample size) was decided based on the microscope time availability.
Data exclusions	As described in the methods section, in line with standard cryoEM data analysis practices, micrographs that showed low CTF estimation resolution or visibly with artifacts arising from protein aggregation, ice contamination or with no single particles were excluded from data analysis.
Replication	Cryo-EM dataset contains several hundred thousand copies of the E1-RFJ complex that therefore shows inherent replication of data points. In addition, randomisation of particle subsets during analysis that showed replication capacity of the data set (i.e.in 2D and 3D analysis) excluded the notion of running multiple experiments.
Randomization	Random particle subsets were used during 3D refinement steps, as implemented in cryoSPARC.
Blinding	No blinding was required based on analysis of random particle subsets.

Reporting for specific materials, systems and methods

We require information from authors about some types of materials, experimental systems and methods used in many studies. Here, indicate whether each material, system or method listed is relevant to your study. If you are not sure if a list item applies to your research, read the appropriate section before selecting a response.

Materials & experimental systems

n/a	Involved in the study	
☒	☐	Antibodies
☒	☐	Eukaryotic cell lines
☒	☐	Palaeontology and archaeology
☒	☐	Animals and other organisms
☒	☐	Human research participants
☒	☐	Clinical data
☒	☐	Dual use research of concern

Methods

n/a	Involved in the study	
☒	☐	ChIP-seq
☒	☐	Flow cytometry
☒	☐	MRI-based neuroimaging
